# A Multidimensional Data Fusion Method Incorporating a Temporal Feature for Estimating Tomato Aboveground Biomass

**DOI:** 10.3390/s26206563

**Published:** 2026-10-16

**Authors:** Fahui Yuan, Rongtao Li, Yong He, Yufei Liu

**Affiliations:** 1 College of Biosystems Engineering and Food Science, Zhejiang University, Hangzhou 310058, China; fahuiyuan@zju.edu.cn (F.Y.); rtli@zju.edu.cn (R.L.); yhe@zju.edu.cn (Y.H.); 2 Key Laboratory of Spectroscopy Sensing, Ministry of Agriculture and Rural Affairs, Hangzhou 310058, China; 3 Zhejiang Key Laboratory of Agricultural Remote Sensing and Information Technology, Zhejiang University, Hangzhou 310058, China

**Keywords:** aboveground biomass, estimation method, multidimensional data fusion, ensemble learning, temporal feature

## Abstract

Aboveground biomass (AGB) serves as a key indicator of tomato seedling growth status and provides a critical basis for guiding precision management. However, existing AGB estimation methods are typically confined to single-time-point assessments and fail to account for phenotypic variations across different growth ages. To address this limitation, this study proposes a growth-stage-aware estimation method for tomato seedling AGB utilizing cross-sectional data collected across multiple growth stages. The proposed method constructs multidimensional input variables from stem phenotypic traits, RGB vegetation indices, and image texture features, and incorporates plant age (days after sowing) as a temporal feature to capture stage-dependent variations during growth. In this study, Bayesian Ridge Regression (BR), Random Forest (RF), and eXtreme Gradient Boosting (XGBoost) were integrated into an ensemble learning framework to leverage the complementary strengths of these models. The proposed stacking ensemble approach yields improved estimation performance, showing potential to mitigate overfitting effects and prediction bias compared with single base models under this evaluation setting. The method achieved an R^2^ of 0.8559 and an RMSE of 0.0363 for tomato AGB estimation. This study provides a novel technical strategy for accurate and multidimensional growth status estimation of greenhouse tomato seedlings.

## 1. Introduction

Tomato is a high-value crop of significant global economic importance [1,2,3]. The growth status at the seedling stage fundamentally determines its yield potential, fruit quality, and adaptability to complex environments [4,5]. Implementing precise interventions during this critical period can significantly improve water and nutrient use efficiency while increasing the economic returns of greenhouse production. Aboveground biomass (AGB) integrates information on seedling growth and overall plant health [6], and serves as a reliable metric for evaluating seedling quality, thereby providing a quantitative basis for precision greenhouse operations [7]. Because traditional destructive measurement is time-consuming, labor-intensive, and unable to capture stage-dependent variations during growth, modern AGB monitoring has shifted toward indirect estimation driven by phenotypic traits [8].

In recent years, machine learning has demonstrated considerable potential for crop AGB and yield prediction, owing to its strong nonlinear fitting capacity [9,10,11]. A growing body of research has validated the effectiveness of combining machine learning with phenotypic traits for AGB estimation. Recent studies have confirmed that the plant height and morphological traits of tomato and eggplant extracted from unmanned aerial vehicle (UAV) images are highly correlated with their actual AGB [12]. By combining machine learning algorithms such as random forest (RF) and artificial neural network (ANN) with phenotypic traits extracted from UAV remote sensing images, high-precision estimation of the AGB and yield of tomato and eggplant was achieved [13,14]. Furthermore, recent advancements in proximal sensing have demonstrated the high reliability of RGB-based non-destructive assessment. For instance, coupling RGB image indices with ANN models has proven highly effective for accurately predicting the AGB, as well as the overall yield of tomatoes under varying environmental conditions [15].

Given the limited characterization capacity of a single sensor, the fusion of multi-source heterogeneous data has emerged as a leading trend in plant phenotyping research [16,17,18]. Researchers have extensively investigated the synergistic integration of multidimensional data in recent years. For instance, Johansen et al. [19] employed a combination of UAV-based morphometric and spectral phenotyping to monitor wild tomato responses to salinity, identifying high-yielding and salt-tolerant accessions through assessments of plant area, growth rate, condition, and projective cover. Lu et al. [20] integrated RGB, multispectral, and thermal infrared data, proposing a volume-density-vitality framework that achieved accurate AGB estimation via the RF algorithm. In addition, Zhang et al. [21] focused on leaf-level indicators, such as leaf area and color indices, and applied machine learning to predict yield, vitamin, and nitrogen nutrition status. These studies have shown that the integration of multidimensional data substantially enhances the performance for tomato growth monitoring and AGB assessment compared to single-source models.

However, the vast majority of existing tomato AGB estimation methods remain confined to static and single-time-point feature extraction, unable to capture stage-dependent variations during growth. This limitation inherently constrains the upper bound of model accuracy, especially under complex background interference. Unlike genuine temporal monitoring studies that longitudinally track the identical individual plants via fixed sensor arrays [22,23], this study leverages phenotypic traits and image texture features across successive developmental phases. This growth-stage-informed approach captures the population’s dynamic accumulation trajectory while accommodating the destructive nature of AGB sampling. In addition, certain machine learning models may face limited interpretability due to their black-box nature. Against this background, this study proposes a multidimensional data fusion method based on a temporal feature. The proposed method builds upon manually measured phenotypic traits of tomato stems and integrates vegetation indices and texture features extracted from RGB images. By leveraging the complementary strengths of multidimensional features and incorporating a temporal feature, this method aims to overcome the limitations of background interference and poor interpretability, and achieves accurate and multidimensional growth status estimation of greenhouse tomatoes during the seedling stage.

## 2. Materials and Methods

### 2.1. Dataset Construction

The experiment was conducted using the tomato cultivar Beiying, grown under standard greenhouse management at the Wuwangnong Group Research Center in Hangzhou, China. Peat was used as the cultivation substrate, and tomato seeds were sown in standard 72-cell plug trays. The trays were then uniformly transferred to an artificial climate chamber for germination and early seedling growth. The chamber was maintained at 28 °C and 65% relative humidity during the daytime period (08:00–20:00, 12 h) and at 18 °C and 55% relative humidity during the nighttime period (20:00–08:00 the following day, 12 h). At 21 days after sowing, 125 uniformly growing tomato seedlings were selected from a total of 144 seedlings across two plug trays and transplanted into the greenhouse plot, with an inter-plant spacing of 0.4 m and a row spacing of 0.7 m. Throughout the experiment, the greenhouse was maintained at an average daytime temperature of 26 °C and an average nighttime temperature of 15 °C. A compound fertilizer (N-P_2_O_5_-K_2_O, 15-15-15) was applied through an integrated water and fertilizer drip irrigation system to ensure adequate water and nutrient supply throughout the growth period, and uniform agronomic management practices were applied to all plants.

Data collection was carried out every four days from 38 to 54 days after sowing, resulting in five sampling batches, with 25 plants destructively sampled per batch. All collected samples were randomly divided, with 70% used as the training set and the remaining 30% reserved as an independent validation set. This random data splitting strategy ensures that the training set captures representative biological variations across all considered developmental phases, allowing the model to learn a global mapping for the entire seedling stage while accounting for individual plant heterogeneity within the same batch.

**Figure 1:**
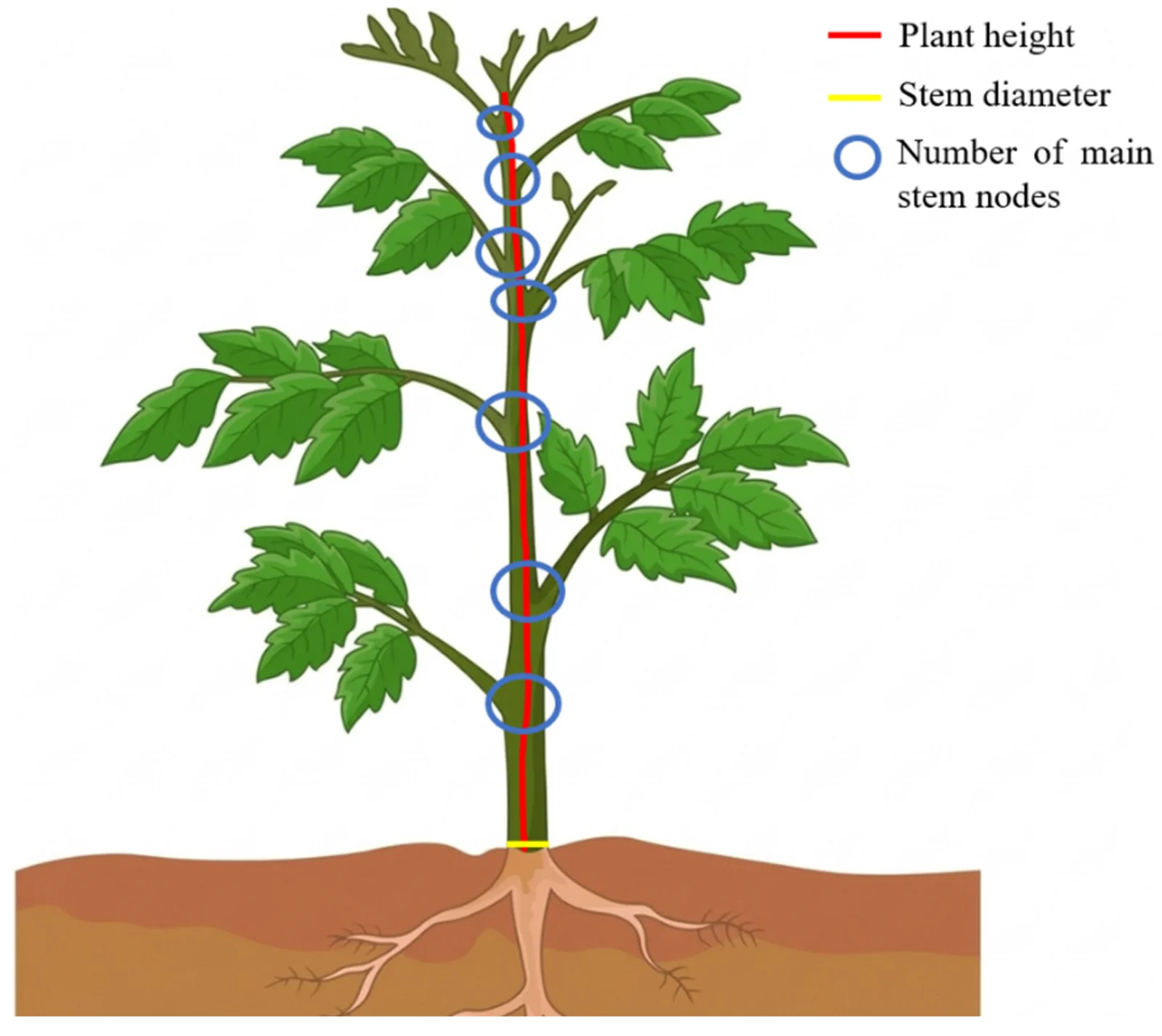
Schematic diagram of stem phenotypic traits.

This study integrates stem phenotypic traits, RGB-based vegetation indices and image texture features to develop a tomato AGB estimation model. As illustrated in Figure 1, the stem phenotypic traits reflect distinct aspects of plant growth: plant height indicates overall developmental progression; stem diameter is closely associated with vascular bundle development; and the number of main stem nodes records the iterative construction of phytomeres [24,25,26]. From the RGB images, vegetation indices capture photosynthetic physiological activity and nitrogen nutritional status, while texture features characterize the structural heterogeneity of the tomato canopy. Collectively, these parameters synergistically describe the growth status of tomato plants and play an indispensable role in AGB estimation models. Table 1 summarizes the agronomic definitions and measurement methods of the stem phenotypic traits mentioned above.

**Figure 2:**
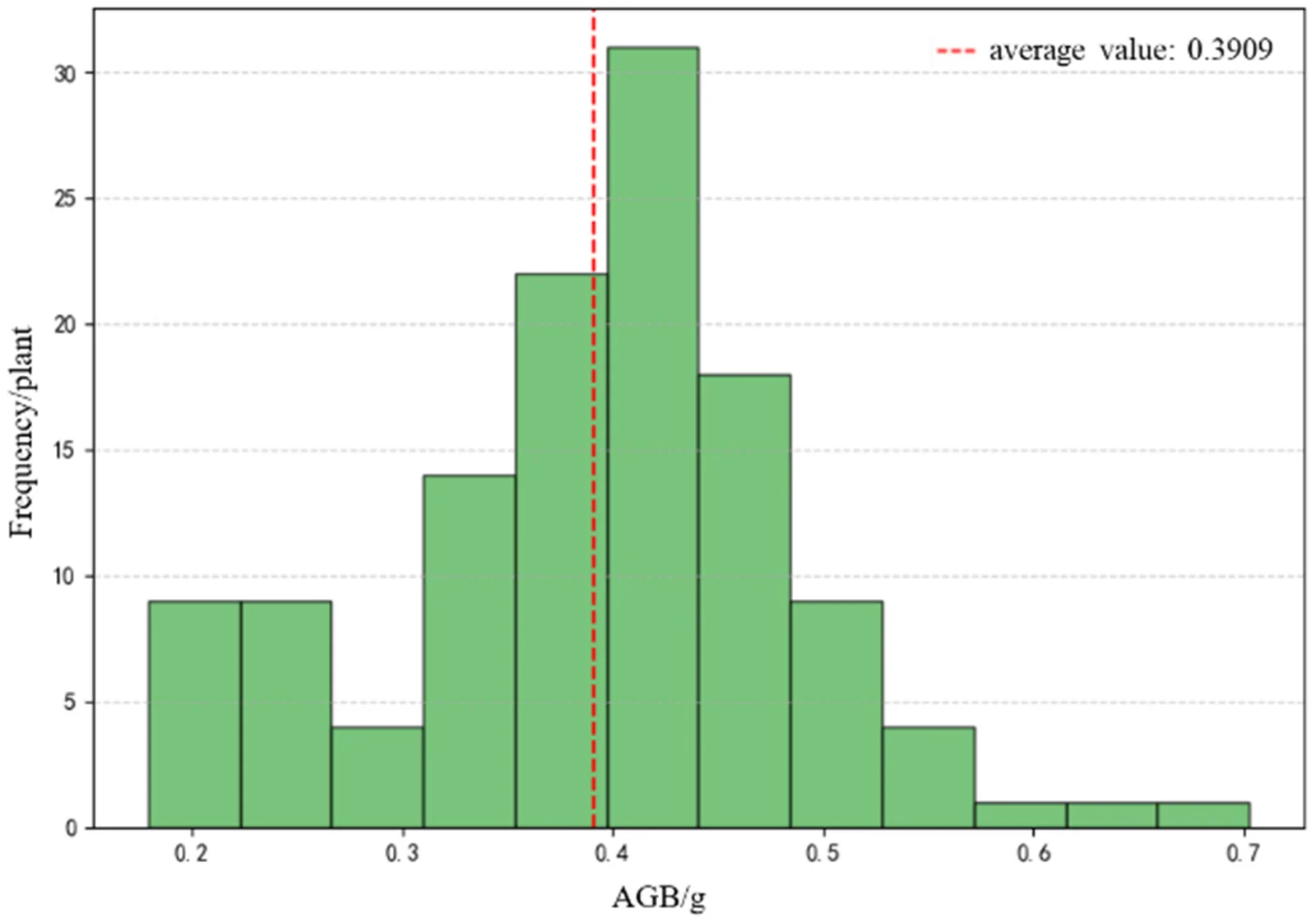
Histogram of the true values of tomato AGB.

RGB images were acquired using a smartphone (Xiaomi 13, Beijing Xiaomi Technology Co., Ltd., Beijing, China). The device was operated in professional mode to capture images in RAW format, with ISO fixed at 100, shutter speed at 1/1000 s, and white balance set to 5000 K. The smartphone was mounted 40 cm above the ground surface, facing vertically downward to ensure nadir image acquisition. Images were acquired during the daytime under similar greenhouse lighting conditions. The tomato AGB dataset generated in this study comprises the dry weight measurements obtained from destructive sampling. To determine the reference AGB, each sampled plant was first heated at 105 °C for 15 min to deactivate enzymes, and then oven-dried at 80 °C until a constant weight was achieved. The dry weight was subsequently measured using an electronic balance with a readability of 0.0001 g. Based on the 125 individual plant samples, the measured AGB values ranged from 0.1536 g to 0.7024 g, with a mean of 0.3909 g and a standard deviation of 0.0994 g. The distribution of these ground-truth AGB values is presented as a histogram in Figure 2.

### 2.2. Acquisition of RGB Image Features

**Figure 3:**
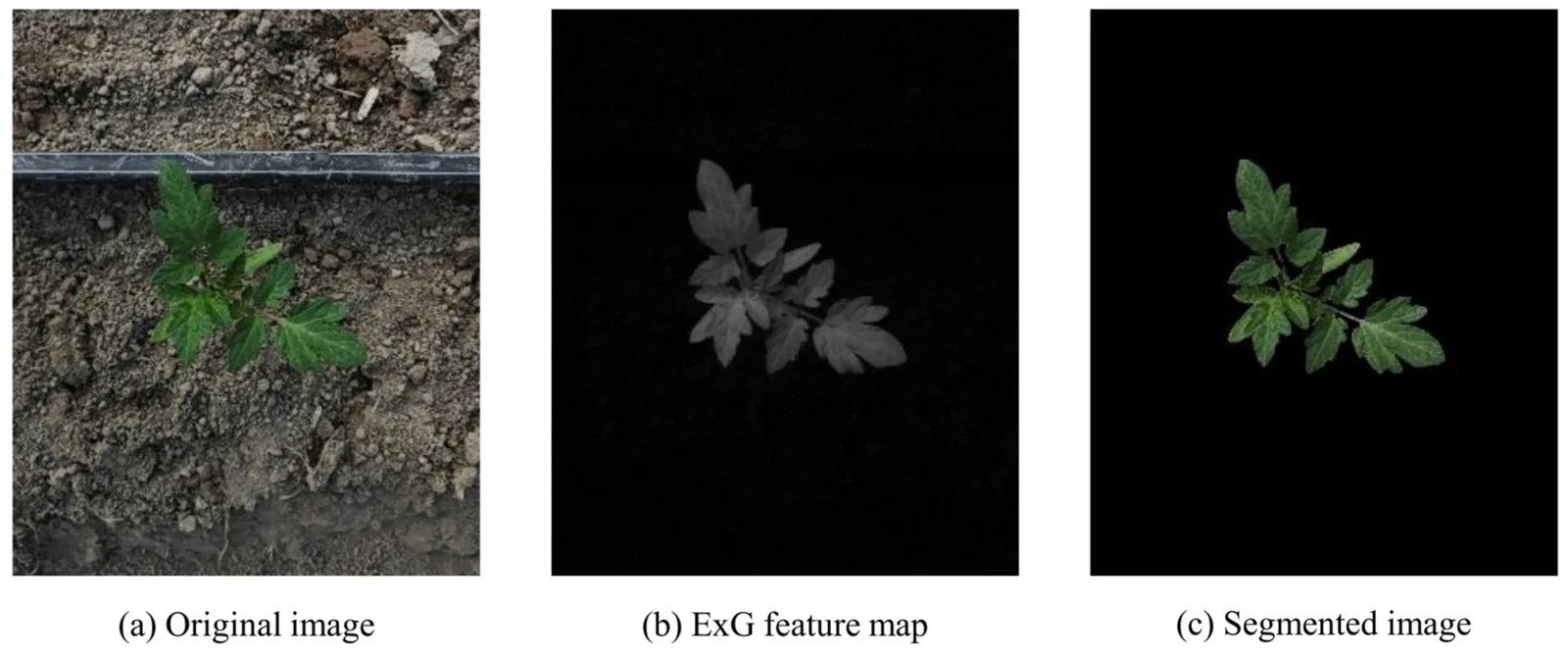
Threshold segmentation results.

To mitigate the effects of uneven illumination, original pixels (R, G, B) were normalized into chromaticity coordinates (r, g, b) before calculating vegetation indices, as detailed in Table 2. The original RGB images contain background objects such as soil, drip irrigation tubes, and weeds, which should be removed to isolate the target plants. However, because moist soil and shaded leaves exhibit similar grayscale intensities, conventional thresholding based on raw pixel values fails to separate them effectively. To address this, the Excess Green (ExG) vegetation index was introduced to enhance the contrast between plant and non-plant regions. The resulting ExG image was then segmented using the Otsu adaptive global thresholding method, which determines the optimal threshold by maximizing the inter-class variance between the foreground and the background. The segmentation results are shown in Figure 3. Visual inspection confirmed that segmentation quality remained robust across all five stages.

RGB-based vegetation indices and image texture features can effectively characterize the growth status of the plant canopy. The synergistic integration of multiple RGB-derived features has become increasingly prevalent in tomato AGB estimation. In this study, commonly used features extracted from the threshold-segmented images were employed to construct the tomato AGB estimation model. The corresponding mathematical expressions are summarized in Table 2 [29,30,31].

### 2.3. Feature Variable Selection

**Figure 4:**
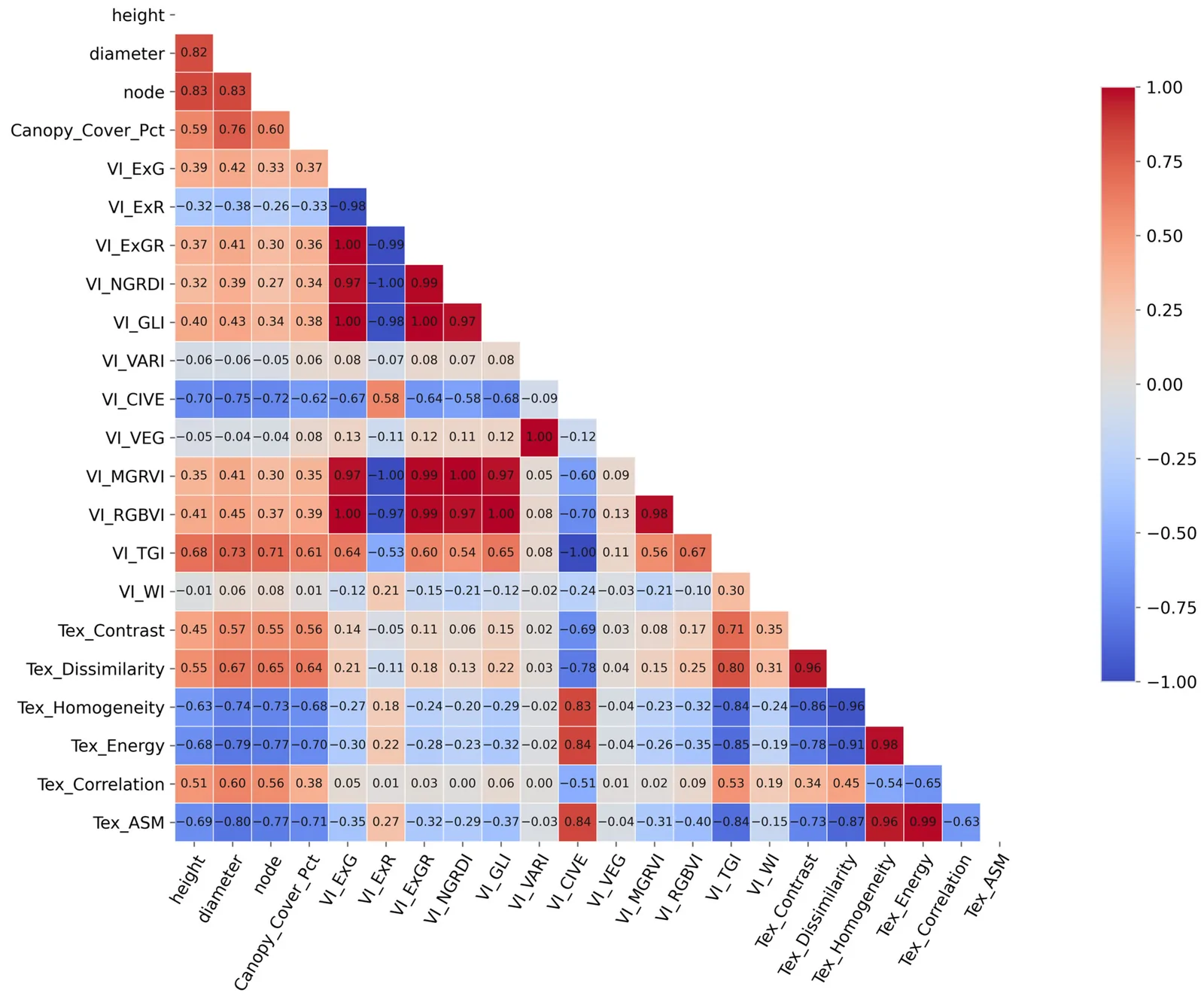
Results of Pearson correlation analysis.

In feature mining, high-dimensional features often exhibit strong linear correlations. Pearson correlation analysis serves as a classical approach for identifying and eliminating redundant features. When two features are highly correlated (|r| > 0.9), the statistical information they convey overlaps substantially. Thus, retaining either one can capture the majority of the information provided by the other. In addition to correlation analysis, significance testing was conducted by calculating the corresponding *p*-values to evaluate the statistical significance of the identified correlations. Correlations with *p* < 0.05 were considered statistically significant, with significance levels denoted as * (*p* < 0.05), ** (*p* < 0.01), and *** (*p* < 0.001). The results of the Pearson correlation analysis performed in this study are presented in Figure 4.

The variables ExGR and ExG exhibited an extremely high correlation (r = 0.9972), indicating severe multicollinearity. Thus, ExGR was removed to improve model stability and reduce the risk of overfitting. Following this principle, among the 22 initial feature variables, 12 redundant ones were eliminated through Pearson correlation analysis, leaving 10 representative feature variables with reduced multicollinearity.

**Figure 5:**
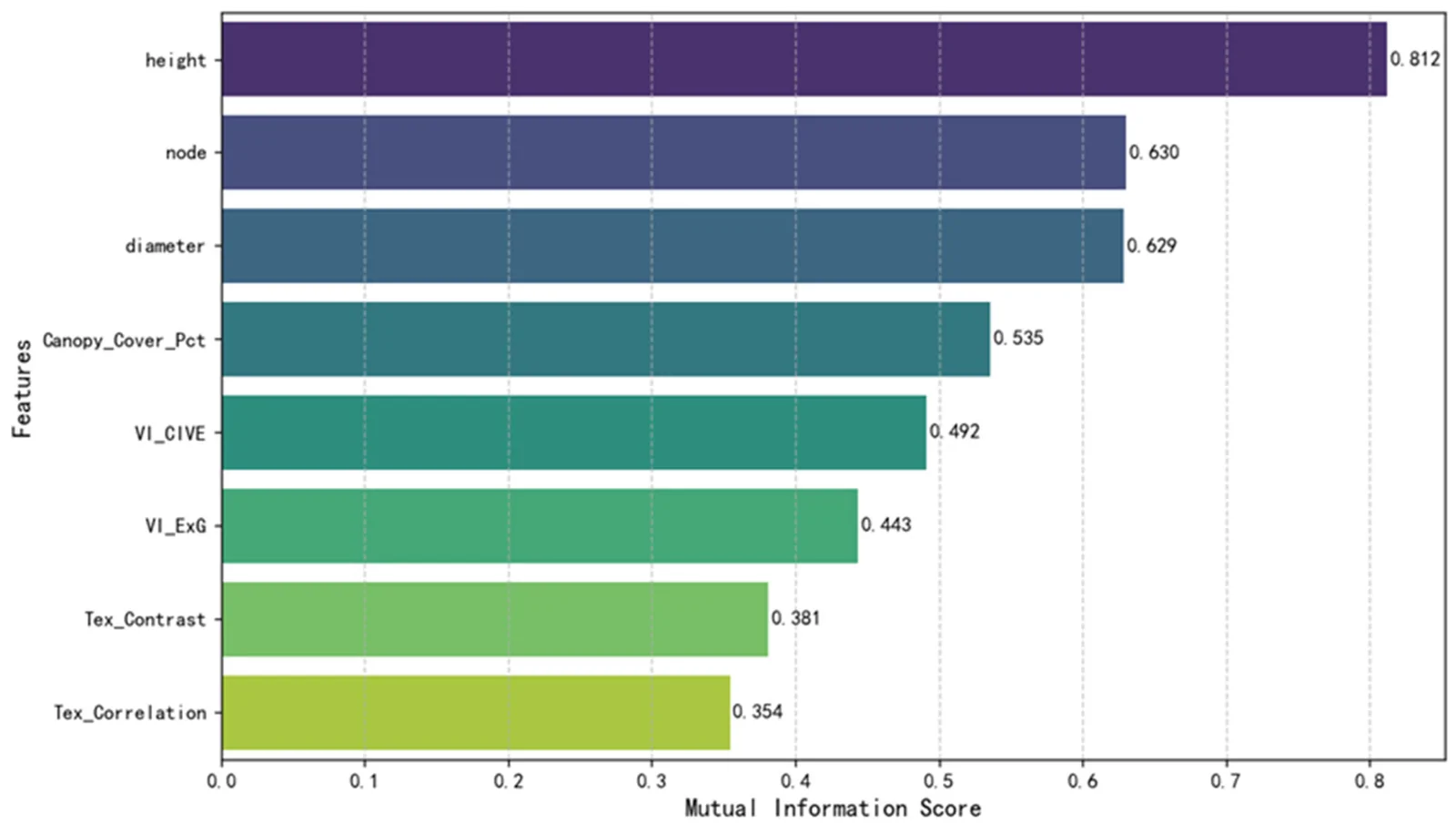
The MI score of eight high-value characteristic variables.

While Pearson correlation analysis can effectively eliminate redundant variables and mitigate multicollinearity, the retained representative feature variables do not necessarily possess strong predictive power for the target variable. Moreover, the Pearson coefficient only captures linear relationships and cannot detect complex nonlinear dependencies. In contrast, mutual information (MI) can capture both linear and arbitrary nonlinear associations, effectively quantifying the interdependence between variables. It plays a crucial role in removing data redundancy and thereby improving the overall predictive performance of the model. To select the most informative features, an empirical threshold of MI > 0.3 was applied. Based on this threshold, eight high-value feature variables were ultimately selected, as illustrated in Figure 5.

### 2.4. Scientific Analysis of Temporal Feature

Tomato seedlings exhibit pronounced allometric growth, meaning that the relative growth rates of different organs vary across developmental stages. Traditional static regression models assume a single and fixed allometric coefficient, thereby neglecting the shifts in resource allocation strategies that occur as plants progress through successive stages. Incorporating the temporal feature allows the model to dynamically adjust the mapping relationship between morphological parameters and AGB according to seedling age. This could reduce the prediction residuals caused by stage-dependent growth variation and enhances the model’s generalization capability in the temporal dimension. Due to the destructive nature of AGB measurements, it is physically impossible to continuously monitor the actual AGB of the same individual plant. Therefore, the temporal feature referred to in this study represents the chronological growth trajectory of the tomato seedling population across multiple growth stages, rather than the continuous tracking of individual plants.

**Figure 6:**
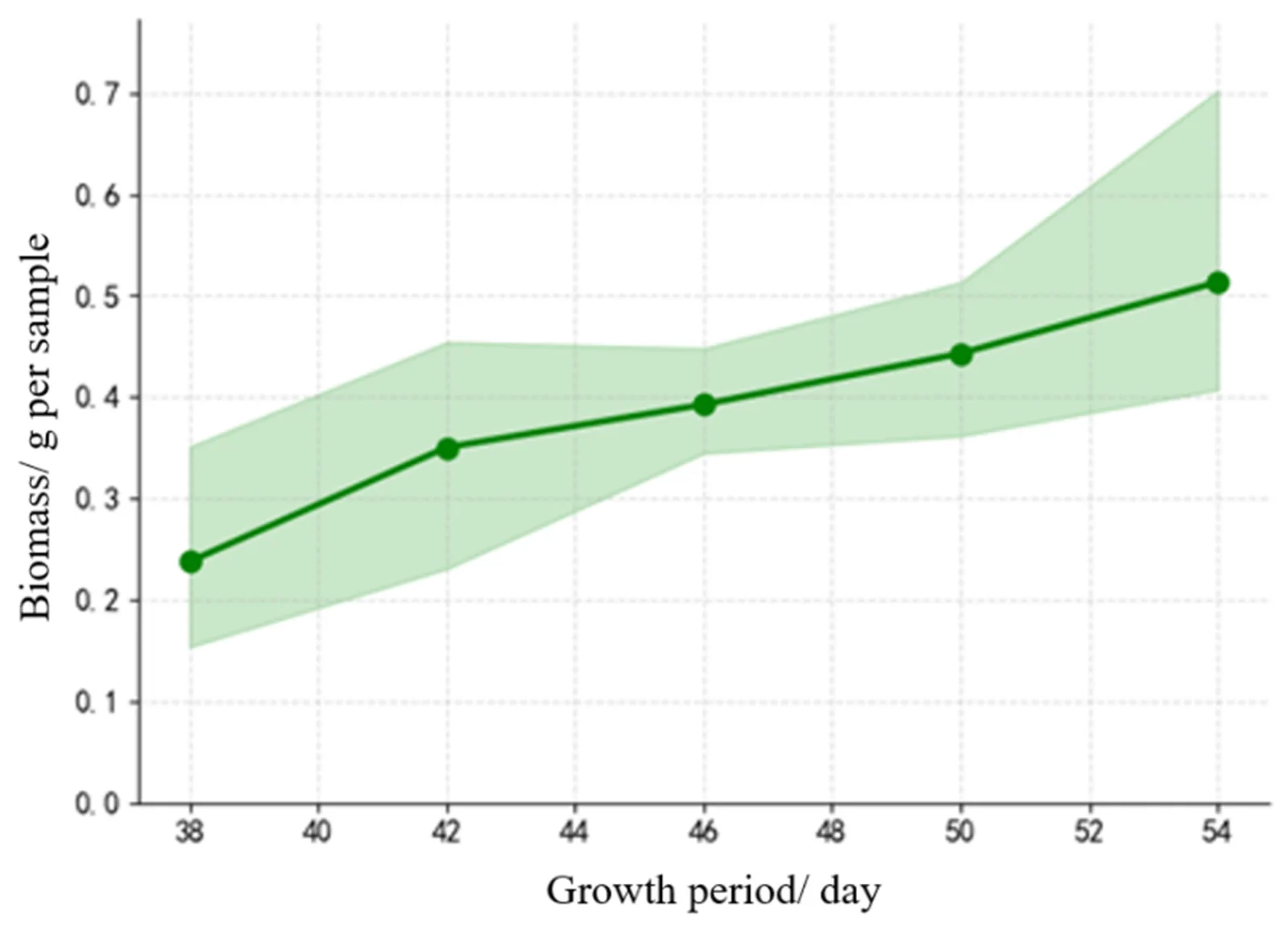
The accumulation process of tomato AGB over time.

The temporal dynamics of tomato AGB estimation are presented in Figure 6. The growth rate was highest between 38 and 42 days after sowing, which was likely attributable to the predominance of morphological development during this period. From 42 to 50 days after sowing, growth slowed, possibly due to the depletion of seed energy reserves and the incomplete establishment of the photosynthetic apparatus in true leaves, resulting in a temporary energy deficit. From 50 to 54 days after sowing, the increase in growth rate may relate to the emergence of more fully expanded true leaves, increased leaf area, and enhanced photosynthetic capacity. These observations indicate that the temporal feature has the potential to serve as a valuable input for AGB estimation models. Accordingly, the number of days after sowing was incorporated as an independent variable in the model. The final tomato AGB estimation model was constructed by integrating multidimensional data, including stem phenotypic traits, vegetation indices, texture features, canopy coverage, and the temporal feature.

### 2.5. Ensemble Learning Model

To address the limited generalization capability and feature-specific sensitivity of single models in tomato AGB estimation, this study developed a stacking-based ensemble learning model. The model adopts a hierarchical two-layer architecture designed to exploit the substantial algorithmic differences among heterogeneous base learners, thereby achieving comprehensive capture of the complex mapping relationships between feature variables and AGB.

Layer 1: Construction of differentiated base learners. The first layer aims to maximize the diversity and breadth of information extraction by incorporating algorithms with distinct inductive biases. Based on their underlying principles, three highly complementary algorithms were selected: Bayesian Ridge Regression (BR), Random Forest (RF), and eXtreme Gradient Boosting (XGBoost). These base learners model the data through three complementary paradigms: linear regularization, nonlinear ensemble partitioning, and iterative residual correction. Specifically, BR employs Bayesian inference with ridge regularization to extract robust linear trends while mitigating multicollinearity among the RGB-derived features; it provides a stable linear foundation for integration. RF constructs randomized decision trees via bootstrap and random subspace sampling, generating complex nonlinear decision boundaries to reduce variance. XGBoost iteratively fits the residuals using a gradient boosting strategy that leverages second-order Taylor expansion of the loss function and explicit regularization, thereby further improving accuracy and reducing residual bias. To ensure model reproducibility and prevent overfitting on the relatively small dataset, the hyperparameters for all base learners were systematically tuned using a grid search strategy combined with five-fold cross-validation on the training set. Specifically, the training dataset was divided into five folds; each base learner was trained on four folds and generated out-of-fold predictions for the remaining validation fold. These out-of-fold predictions were then concatenated to form the input feature matrix for training the second-layer meta-learner.

Layer 2: Adaptive meta-learner fusion. To avoid the information loss typically associated with simple averaging, ridge regression was introduced as the meta-learner. The predictions ($\hat{y_{BR}},\hat{y_{RF}},\hat{y_{XGB}}$) from the three base learners were extracted and stacked to form a new feature matrix for the second layer. Because different base learners perform unevenly across sample sub-spaces and their predictions tend to be highly correlated, ridge regression can effectively manage this collinearity among the meta-features. By learning optimal regression coefficients, the meta-learner achieves an adaptive weighted fusion of the base model outputs, yielding the final AGB estimate. Specifically, the weight learning problem of the meta-learner was modeled as a convex optimization problem incorporating an L2 regularization term. The objective loss function was given by the sum of the squared residuals and the L2 penalty, expressed as:

(1) $$J\left(\alpha \right)={\left|\left|y-Z\alpha \right|\right|}_{2}^{2}+\lambda {\left|\left|\alpha \right|\right|}_{2}^{2}$$

where Z is the feature matrix formed by the prediction results of the first-layer base-learners, $\alpha ={\left[\begin{array}{ccc} \alpha_{1} & \alpha_{2} & \alpha_{3} \end{array}\right]}^{T}$ represents the adaptive weight parameters, λ is the regularization coefficient used to control model complexity and suppress multicollinearity among the base-learners predicted values, and *y* is the measured AGB. λ was initially selected empirically as a standard default and subsequently verified via cross-validation, confirming that setting to 1. Because this objective function is convex, the global optimal solution can be obtained by taking the derivative with respect to *α* and setting it to zero.

(2) $$\alpha^{*}={\left(Z^{T}Z+\lambda I\right)}^{-1}Z^{T}y$$

(3) $$\hat{Y_{final}}=\alpha_{1}f_{BR}\left(X\right)+\alpha_{2}f_{RF}\left(X\right)+\alpha_{3}f_{XGB}\left(X\right)+\epsilon$$

where *I* is a 3 × 3 identity matrix, *X* is the original multidimensional input feature matrix fed into the base models, and ε represents the intrinsic bias or residual error term of the meta-learner.

By integrating linear, nonlinear, and iterative residual correction mechanisms, the proposed ensemble model achieves complementary learning across heterogeneous base learners. Through the regularization imposed by the ridge meta-learner, the model avoids over-reliance on any single base learner, thereby reducing the overall generalization error. In summary, this study leveraged multidimensional feature fusion within a stacking-based ensemble framework to perform high-precision estimation of tomato AGB.

## 3. Results and Discussion

### 3.1. Estimation Results of Empirical Models

Empirical models establish functional relationships between feature variables and tomato AGB by fitting historical data, and they retain considerable practical value in small-dataset scenarios. Accordingly, this study employed four classical regression models for tomato AGB estimation: linear, polynomial, exponential, and logarithmic, expressed as follows:

Linear regression model:

(4) y=ax+b

Polynomial regression model:

(5) $$y={ax}^{2}+bx+c$$

Exponential regression models:

(6) $$y=ab^{x}$$

Logarithmic regression models:

(7) y=alogx+b

where *y* is the tomato AGB value in the regression model, *x* represents the feature variables used for model construction, and a, b, and c are regression model coefficients. All empirical and machine learning models were evaluated using the exact same 30% independent validation subset to ensure a fair and direct comparison. The estimation results of the empirical models are shown in Table 3.

The results presented in Table 3 indicate that, among the evaluated features, the polynomial model achieved the best overall performance. When plant height was used as the single predictor, the polynomial model yielded the highest R^2^ (0.7971) and the lowest RMSE (0.0454). In contrast, the model based on ExG performed poorly, with an R^2^ of only 0.3015. This discrepancy can be attributed to the fact that, during the tomato seedling stage, stem-related traits dominate the total AGB, whereas the contribution of canopy characteristics remains relatively small. Overall, the predictive accuracy of the empirical models was limited, underscoring the need for more advanced estimation methods to improve tomato AGB estimation in small-sample scenarios.

### 3.2. Estimation Results of Machine Learning and Ensemble Learning Models

**Figure 7:**
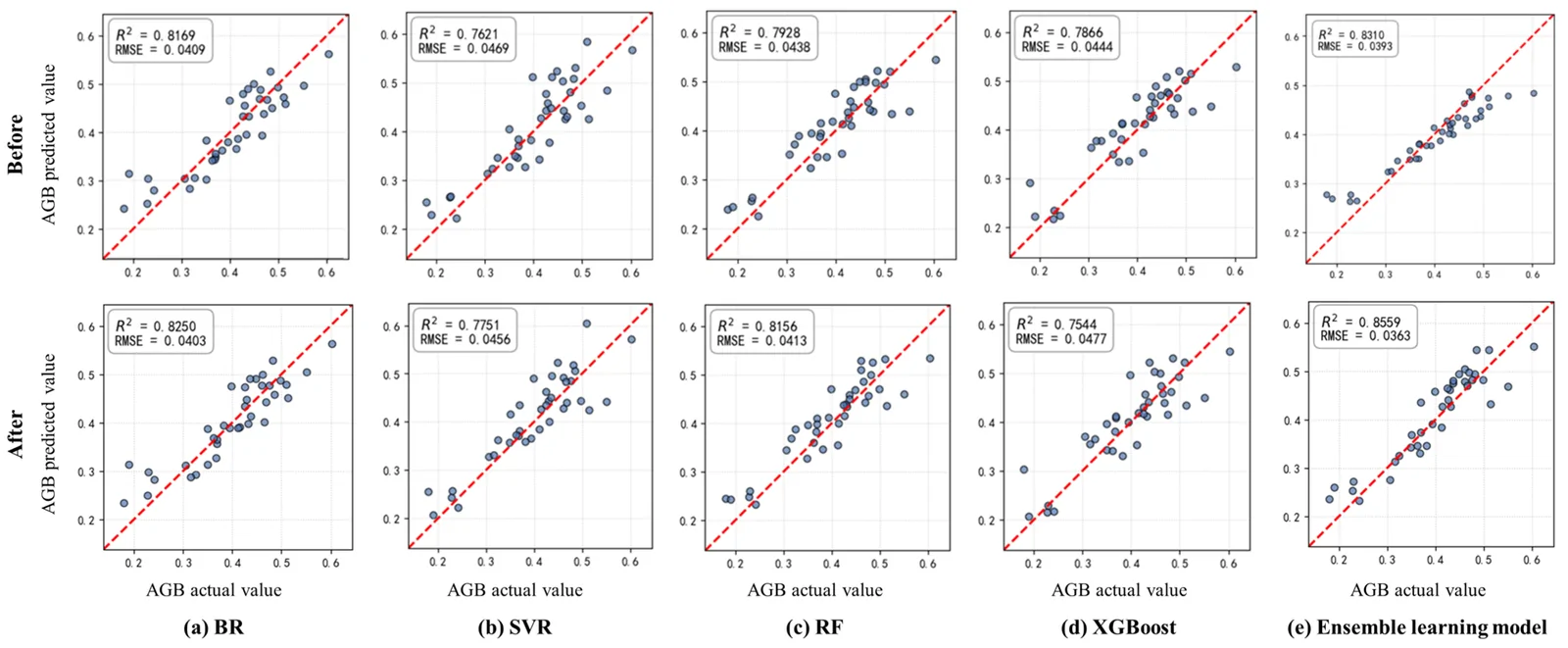
Scatter plot of the estimation results of five models before and after incorporating the temporal feature.

To evaluate the performance of the proposed ensemble model, four standalone machine learning regression algorithms (BR, SVR, RF, and XGBoost) were also applied to tomato AGB estimation, and their results were compared with those of the ensemble method. The scatter plots of measured versus predicted AGB values for all five algorithms are presented in the upper half of Figure 7, providing a direct visual assessment of their fitting performance.

The scatter plots indicate that, among the four standalone machine learning models, BR achieved the highest R^2^ (0.8169) and the lowest RMSE (0.0409) for tomato AGB estimation, outperforming the traditional empirical models. This superior performance may be attributed to the ability of BR to effectively capture the relationships between feature variables and AGB while constraining overfitting through L2 regularization. In contrast, SVR yielded the least accurate predictions, possibly because it struggles to construct reliable high-dimensional hyperplanes when trained on limited samples. Despite these improvements, individual machine learning models still face challenges in fully capturing the complex relationships between the input features and AGB.

The proposed stacking-based ensemble model produced predicted values that were in strong agreement with the measured AGB, achieving an R^2^ of 0.8310 and an RMSE of 0.0393. By integrating the complementary strengths of the three base learners and using a ridge meta-learner to adaptively fuse their outputs, the ensemble method effectively reduced prediction bias and improved overall accuracy. However, for samples with AGB values exceeding 0.5 g, the model’s extrapolation capability declined, possibly because such samples accounted for only 4% of the total samples, leading to increased prediction errors. Future work will extend the sampling period to cover a wider range of growth stages and increase the representation of high-biomass samples, thereby overcoming the current data limitations. It should be noted that the camera was fixed at 40 cm above the ground, while the distance between the camera and the canopy decreased as the plants grew. This may introduce scale variation in canopy coverage and texture features. Although the temporal feature and multidimensional features partially captured stage-dependent variations during growth, future work should consider incorporating distance-based normalization to further improve feature consistency and model robustness.

### 3.3. Effect of Temporal Feature on AGB Estimation Performance

In this study, the temporal feature of days after sowing was incorporated as an independent variable. The contribution of this temporal variable was assessed by comparing the prediction performance of the ensemble model with and without its inclusion. The estimation results after adding the temporal feature variable are presented in the lower half of Figure 7.

As shown in Figure 7, most models exhibited varying degrees of improvement in prediction performance after incorporating the temporal feature, with the proposed ensemble learning model achieving the largest gain, reaching an R^2^ of 0.8559 and an RMSE of 0.0363. Notably, the addition of a temporal feature improves discriminative power during high-biomass stages, thereby enabling more reliable predictions under conditions where static models typically saturate. In contrast, XGBoost unexpectedly degraded, likely because its gradient boosting strategy overfitted to the dominant large residuals of later growth stages, misweighting the subtler morphological patterns in early and middle stages. Overall, the temporal feature positively contributed to tomato AGB estimation for the majority of models and can be effectively used as a predictor variable. This study demonstrates that the proposed ensemble learning framework, which integrates the temporal feature with multidimensional data, enables accurate estimation of tomato AGB and robust growth monitoring.

## 4. Conclusions

This study proposes an ensemble learning model that integrates a temporal feature with multidimensional data for tomato AGB estimation. The model employs BR, RF, and XGBoost as base learners within an ensemble framework to leverage their complementary strengths, while incorporating a temporal feature to capture stage-dependent variations during growth. By integrating stem phenotypic traits, RGB-derived vegetation indices, and texture features, the model achieves an R^2^ of 0.8559 and an RMSE of 0.0363 for AGB estimation, enabling accurate growth monitoring of tomatoes during the investigated seedling stage. Despite the successful establishment of this core predictive mapping, certain limitations must be acknowledged. For example, the current reliance on manual measurements for stem traits restricts the model’s large-scale applicability. Future work will focus on integrating computer vision technologies to automate phenotypic data acquisition. Furthermore, while the proposed method accurately estimates AGB, its performance was currently validated within a restricted experimental scope. Therefore, future research must prioritize external validation across independent planting batches, diverse cultivars, and extended developmental stages. Subsequent studies will also aim to expand the dataset to strictly isolate feature selection from model training, ultimately enhancing the model’s temporal extrapolation capabilities across unseen growth phases.

## Figures and Tables

**Table 1:** The agronomic definitions and measurement methods of stem phenotypic traits.

Trait	Definition	Measurement Method	Reference
Plant height	The length from the stem base to the apical growing point	Measure three times with a flexible tape measure to obtain an average, with a minimum unit of 0.1 cm	[27]
Stem diameter	The width of the stem at the base	Measure three times using a vernier caliper to calculate an average, with a minimum unit of 0.02 mm	[24]
Number of main stem nodes	The total number of nodes on the main stem of the plant from the base to the top	Count visually, with a minimum unit of 1 node	[28]

**Table 2:** RGB image features and calculation formulas.

No.	Feature	Expression Formula	No.	Feature	Expression Formula
1	Canopy Cover	$\mathrm{CC}=\frac{N_{\mathrm{plant}\_\mathrm{pixels}}}{N_{\mathrm{total}\_\mathrm{pixels}}}\times 100\%$	11	Red-Green-Blue Vegetation Index	$\mathrm{RGBVI}=\frac{g^{2}-r\cdot b}{g^{2}+r\cdot b}$
2	Excess Green	ExG=2g−r−b	12	Triangular Greenness Index	TGI=−0.5[190(R−G)−120(R−B)]
3	Excess Red	ExR=1.4r−g	13	Woebbecke Index	$\mathrm{WI}=\frac{g-b}{r-g}$
4	Excess Green minus Excess Red	ExGR=ExG−ExR	14	Contrast	$\mathrm{Contrast}=\sum_{i=0}^{N-1}\sum_{j=0}^{N-1}{\left(i-j\right)}^{2}P\left(i,j\right)$
5	Normalized Green-Red Difference	$\mathrm{NGRDI}=\frac{g-r}{g+r}$	15	Dissimilarity	$\mathrm{Dissimilarity}=\sum_{i=0}^{N-1}\sum_{j=0}^{N-1}\left|i-j\right|P\left(i,j\right)$
6	Green Leaf Index	$\mathrm{GLI}=\frac{2g-r-b}{2g+r+b}$	16	Homogeneity	$\mathrm{Homogeneity}=\sum_{i=0}^{N-1}\sum_{j=0}^{N-1}\frac{P\left(i,j\right)}{1+{\left(i-j\right)}^{2}}$
7	Visible Atmospherically Resistant Index	$\mathrm{VARI}=\frac{g-r}{g+r-b}$	17	Energy	$\mathrm{Energy}=\sqrt{\sum_{i=0}^{N-1}\sum_{j=0}^{N-1}\{P\left(i,j\right){\}}^{2}}$
8	Color Index of Vegetation Extraction	CIVE=0.441r−0.811g+0.385b+18.787	18	Correlation	$\mathrm{Correlation}=\sum_{i=0}^{N-1}\sum_{j=0}^{N-1}\frac{\left(i-\mu_{x}\right)\left(j-\mu_{y}\right)P\left(i,j\right)}{\sigma_{x}\sigma_{y}}$
9	Vegetative Index	$\mathrm{VEG}=\frac{g}{r^{a}b^{\left(1-a\right)}},\left(a\approx 0.667\right)$	19	Angular Second Moment	$\mathrm{ASM}=\sum_{i=0}^{N-1}\sum_{j=0}^{N-1}\{P\left(i,j\right){\}}^{2}$
10	Modified Green Red Vegetation Index	$\mathrm{MGRVI}=\frac{g^{2}-r^{2}}{g^{2}+r^{2}}$			

**Table 3:** Estimation results of the empirical model.

Characteristic Variable	Expression Formula	R^2^	RMSE
Plant height	$y=-0.0008x^{2}+0.0518x+0.0150$	0.7971	0.0454
Stem diameter	$y=-0.0116x^{2}+0.1663x-0.0267$	0.7139	0.0540
Number of main stem nodes	$y=-0.0591x^{2}+0.5097x-0.5899$	0.6680	0.0581
CC	$y=-0.0025x^{2}+0.0690x+0.0637$	0.6064	0.0633
CIVE	$y=-0.0007x^{2}-0.0042x+0.5052$	0.5264	0.0694
ExG	$y=-2.4747x^{2}+1.9070x+0.0530$	0.3015	0.0843
Contrast	$y=-0.0018x^{2}+0.0475x+0.1870$	0.4908	0.0720
Correlation	$y=-352.4361x^{2}+690.2750x-337.5425$	0.3633	0.0805

## Data Availability

The dataset used in this study has been made publicly available on Figshare (https://doi.org/10.6084/m9.figshare.33092837).
